# High-Strength Albumin Hydrogels With Hybrid Cross-Linking

**DOI:** 10.3389/fchem.2020.00106

**Published:** 2020-02-25

**Authors:** Shaoping Lu, Lin Zhu, Qilin Wang, Zhao Liu, Chen Tang, Huan Sun, Jia Yang, Gang Qin, Gengzhi Sun, Qiang Chen

**Affiliations:** ^1^School of Materials Science and Engineering, Henan Polytechnic University, Jiaozuo, China; ^2^Key Laboratory of Flexible Electronics (KLOFE), Institute of Advanced Materials (IAM), Jiangsu National Synergetic Innovation Center for Advanced Materials (SICAM), Nanjing Tech University (NanjingTech), Nanjing, China

**Keywords:** protein hydrogels, high strength, non-swelling, self-recovery, fatigue resistance

## Abstract

Natural protein-based hydrogels possess excellent biocompatibility; however, most of them are weak or brittle. In the present work, high strength hybrid dual-crosslinking BSA gels (BSA DC gels), which have both chemical cross-linking and physical cross-linking, were fabricated by a facile photoreaction-heating process. BSA DC gels showed high transparency (light transmittance of ~90%) and high strength. At optimal conditions, BSA DC gel exhibited high compressive strength (σ_c,f_) of 37.81 ± 2.61 MPa and tensile strength (σ_t,f_) of 0.62 ± 0.078 MPa, showing it to be much stronger than physically cross-linked BSA gel (BSA PC gel) and chemically cross-linked BSA gel (BSA CC gel). More importantly, BSA DC gel displayed non-swelling properties while maintaining high strength in DI water, pH = 3.0, and pH = 10.0. Moreover, BSA DC gel also demonstrated large hysteresis, rapid self-recovery, and excellent fatigue resistance properties. It is believed that our BSA DC gel can potentially be applied in biomedical fields.

## Introduction

Because of excellent biocompatibility, natural protein hydrogels have attracted a great deal of attention and have been used in a wide range of biological and biomedical fields (Koetting et al., 2016; Partlow et al., 2016; Silva et al., 2017; Jo et al., 2018; Gacanin et al., 2019). However, the poor mechanical properties of natural protein hydrogels is one of the main drawbacks impeding their application in some cases (Silva et al., 2017; Tang et al., 2018). Recently, great efforts have been made to enhance the mechanical properties of natural protein hydrogels, and some strategies have been developed, including nanocomposite (Yuk et al., 2016; Qin et al., 2017; Wang et al., 2017, 2018), physical cross-linking (Toivonen et al., 2015; Feng et al., 2018; Yan et al., 2019; Zhang et al., 2019), solvent induction (Li et al., 2016; Zhang et al., 2016; Hashemnejad et al., 2017; Zhu et al., 2018), double network (Bhattacharjee et al., 2015; Luo et al., 2016; Rangel-Argote et al., 2018; Tavsanli and Okay, 2019), hybrid cross-linking (Moura et al., 2011; Epstein-Barash et al., 2012; Xu et al., 2016; Nojima and Iyoda, 2018; Yang et al., 2018), and so on.

Among them, hybrid cross-linking, consisting of chemical and physical cross-linkings, is a simple and effective method of preparing high-performance natural protein hydrogels, especially for silk fibroin (SF) hydrogels. For example, Numata et al. (2017) reported the creation of hybrid SF hydrogels via an enzymatic oxidation reaction to form chemical cross-links between dityrosine residues and phase separation of silk solution to produce physical cross-links, which demonstrated high a compressive strength of ~14 MPa. Okay and coworkers found that SF cryogels showed remarkable properties (compressive modulus of 48 MPa and strength of 970 kPa) if chemical cross-linkers (ethylene glycol diglycidyl ether) were added into the cryogelation system (Yetiskin et al., 2017). Nevertheless, these high strength hybrid natural protein hydrogels are mainly fibril protein hydrogels, and little attention focuses on natural globulin protein hydrogels.

Bovine serum albumin (BSA) is one of the typical natural globulin proteins with a large number of functional groups, which provide many possibilities for chemical cross-linking of BSA to form covalent BSA gels. It is well-known that tyrosine groups can form dityrosine bonds via enzymatic reaction, and these play an important role in the mechanical properties of natural materials (Endrizzi et al., 2006; Partlow et al., 2014; Tavsanli and Okay, 2019). Considering that BSA has 21 tyrosines, it is an ideal template for the design and synthesis of natural globulin protein hydrogels via dityrosine bonds. However, dityrosine bond cross-linked BSA gels may exhibit brittle properties, similar to other gels (Sun and Huang, 2016; Fernandez-Castano Romera et al., 2017; Lü et al., 2017; Kabb et al., 2018; Picchioni and Muljana, 2018).

Herein, high-strength hybrid dual-crosslinking BSA gels (BSA DC gels) were fabricated by covalent cross-linking of BSA via dityrosine bonds followed by heat-induced denaturation of protein to form physical cross-linkings between BSA molecules. BSA is selected in the present work because it has plenty of tyrosine groups, low cost, high water solubility, and good biocompatibility. BSA DC gels showed high transparency and high strength. Under optimal conditions, BSA DC gel exhibited high compressive strength (σ_c,f_) of 37.81 ± 2.61 MPa and tensile strength (σ_t,f_) of 0.62 ± 0.078 MPa, showing it to be much stronger than physically cross-linked BSA gel (BSA PC gel, σ_c,f_ of 3.10 ± 0.06 MPa and σ_t,f_ of 0.06 MPa) and chemically cross-linked BSA gel (BSA CC gel, σ_c,f_ of 2.49 ± 0.29 MPa and σ_t,f_ of 0.25 MPa). More importantly, BSA DC gel displayed non-swelling properties while maintaining high performance in DI water, pH = 3.0, and pH = 10.0. Owing to the presence of physical interactions, BSA DC gel also demonstrated large hysteresis, rapid self-recovery and excellent fatigue resistance properties.

## Results and Discussion

### Network Structure and Morphology

**Figure 1:**
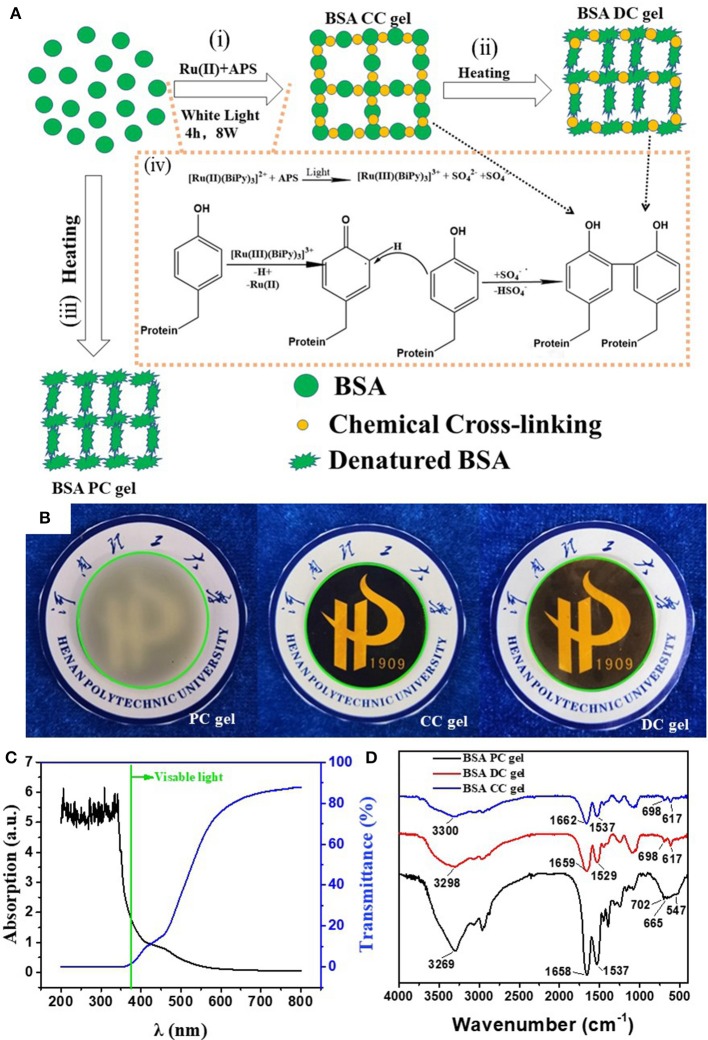
**(A)** Illustration of the preparation and network structure of BSA DC gel; **(B)** appearance of three BSA gels; **(C)** transmittance and adsorption of BSA DC gel; **(D)** FTIR of BSA DC gel, BSA PC gel, and BSA CC gel.

**Figure 2:**
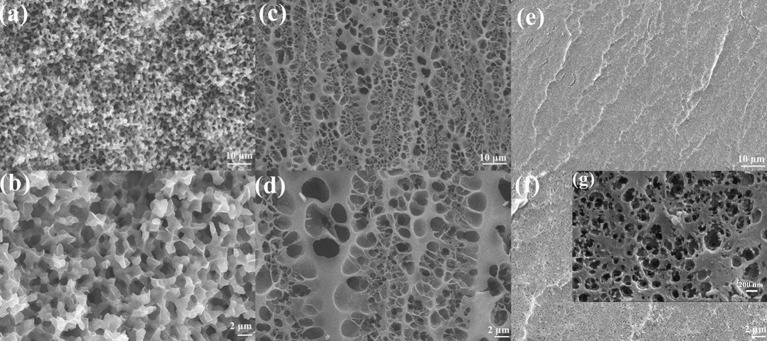
SEM images of **(a,b)** BSA PC gel, **(c,d)** BSA CC gel, and **(e,f,g)** BSA DC gel. Scale bars of **(a,c,e)** are 10 μm, while scale bar of **(b,d,f)** is 2 μm. Scale bar of **(g)** is 200 nm.

As shown in Figure 1A, BSA DC gel was prepared by a mild, simple, and “one-pot” method. First, BSA was chemically cross-linked by dityrosine bonds via ammonium persulfate (APS) and tris[2,2′-bipyridine] ruthenium dichloride [Ru(II)]-induced photoreaction under white light (i.e., BSA CC gel). Then, the gel was heated to induce denaturation of BSA, which formed a physically cross-linked network. BSA PC gel was fabricated via directly heating the BSA solution without the photoreaction. The appearance of the three BSA gels is shown in Figure 1B. BSA CC gel and BSA DC gel were red-brown (Figure 1B), indicating the formation of dityrosine bonds after photoreaction. The gelation mechanism of tyrosine-rich protein via APS/Ru(II) under white light is well-known and is illustrated in Figure 1A (Partlow et al., 2016; Silva et al., 2017). Interestingly, different from BSA PC gel, which had an opaque milk-white color, BSA CC gel and BSA DC gel showed high transparency, and the logo of our university could be clearly seen through them (Figure 1B). The light transmittance of BSA DC gel at visible light range reached up to 90%, while ultraviolet (UV) light could not transmit through the gel and was totally absorbed (Figure 1C). The results indicate that our BSA DC gel might potentially be applied as a UV shielding material. Moreover, a peak at ~460 nm on the UV adsorption spectrum of BSA DC gel was detected (Figure S1), indicating the formation of dityrosine bonds (Ma et al., 2016; Lee et al., 2019). The formation of dityrosine bonds was also characterized by FTIR (Figure 1D). Compared to BSA PC gel, new peaks at 617 and 698 cm^−1^ were distinctly detected for BSA CC gel and BSA DC gel, which were the evidence for dityrosine bonds. Compared to BSA CC gel, the characteristic peak of –OH groups in BSA DC gel was shifted from 3,300 to 3,298 cm^−1^, and the amide I in BSA DC gel was shifted from 1,662 to 1,658 cm^−1^(Militello et al., 2004), indicating that there are physical interactions after heat-induced denaturation. The morphologies of freeze-dried BSA PC gel, BSA CC gel, and BSA DC gel are illustrated in Figure 2. BSA PC gel exhibited a particle-aggregation network structure, which was consistent with our previous report (Tang et al., 2018, Figures 2a,b). BSA CC gel displayed a heterogeneous porous network structure (1–5 μm, Figures 2c,d). Compared to BSA CC gel, BSA DC gel demonstrated a much denser porous network structure, and the pore size was 50–200 nm. The denser network structure of BSA DC gel might be attributable to the higher cross-linking density compared to BSA CC gel.

### Mechanical Properties

**Figure 3:**
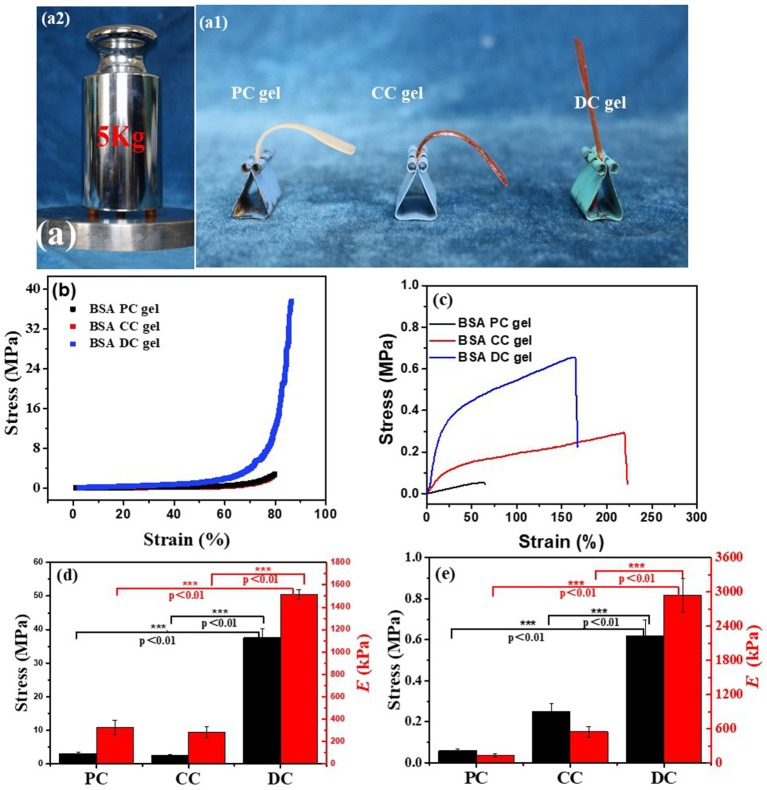
**(a)** BSA DC gel could bear a 5 kg weight without breakage (a_1_), and BSA DC gel could also sustain its own weight without bending, while BSA PC gel and BSA CC gel were bent (a_2_); **(b)** compressive and **(c)** uniaxial tensile stress–stain curves of the three BSA gels; **(d)** compressive modulus and compressive strength and **(e)** tensile modulus and tensile strength of the three BSA gels.

Owing to the hybrid cross-linking network structure, BSA DC gel possessed high strength. Three cylindrical samples (8.5 × 10 mm) of BSA DC gel could withstand 5 kg weight without breakage (Figure 3a_1_). As shown in Figure 3a_2_, BSA DC gel sustained its weight without bending; however, BSA PC gel and BSA CC gel were bent because of their weight. Compressive and tensile tests were conducted to evaluate the mechanical properties of BSA DC gels. From Figure 3b, it can be clearly seen that BSA DC gel exhibited much better compressive properties. Consistently, BSA DC gel showed compressive strength (σ_c_) of 37.81 ± 2.61 MPa, which was 12 and 15 times larger than BSA PC gel (3.10 ± 0.06 MPa) and BSA CC gel (2.49 ± 0.29 MPa) (Figure 3c), respectively. Similarly, the compressive modulus of BSA DC gel was 1515 ± 43 kPa, which was much stiffer than those of BSA PC gel (254 ± 8 kPa) and BSA CC gel (284 ± 48 kPa), respectively. Meanwhile, BSA DC gel displayed high tensile properties (Figure 3d): the tensile strength (σ_f_) was 0.62 ± 0.078 MPa. In contrast, the σ_f_ values of BSA PC gel and BSA CC gel were 0.06 ± 0.008 and 0.25 ± 0.04 MPa, respectively. BSA DC gel also showed a higher tensile modulus (2939 ± 289 kPa), which was the reason that BSA DC gel could sustain its weight without bending. Statistical analysis was also performed (Figures 3d,e). BSA DC gels exhibited significant differences compared to BSA CC gel and BSA PC gel (*p* < 0.01), indicating that hybrid cross-linking is an effective strategy for improving the mechanical properties of BSA gels.

The effects of various parameters, such as BSA concentration (C_BSA_), Ru(II) concentration [C_Ru(II)_], exposure time, heating temperature, and heating time, on the compressive properties of BSA DC gels were also investigated, and the results are summarized in Table S1. The compressive properties increased along with the increase in C_BSA_, i.e., σ_c_ and *E*_c_ increased from 0.54 ± 0.03 to 34.73 ± 2.92 MPa and from 292 ± 17 to 1,320 ± 25 kPa, respectively. At C_BSA_=100 mg/mL, BSA DC gel was too week to test. Compared to the gel at C_BSA_ = 300 mg/mL, the σ_c_ value of the gel at C_BSA_ = 400 mg/mL was significantly increased from 3.22 ± 0.61 to 26.00 ± 2.10 MPa. The highest value reached was 34.73 ± 2.92 MPa at C_BSA_ = 500 mg/mL. The presence of Ru(II) was also important for achieving high-strength BSA DC gels. Without Ru(II), σ_c_ was only 3.10 ± 0.06 MPa. However, after adding a very small amount of Ru(II) (C_Ru(II)_ = 200 μM), σ_c_ reached up to 23.45 ± 1.37 MPa. The compressive properties of BSA DC gel were the best at C_Ru(II)_ = 600 μM (σ_c_ of 34.73 ± 2.92 MPa and *E*_c_ of 1320 ± 25 kPa). Unfortunately, the compressive properties deteriorated if C_Ru(II)_ >600 μM, probably because of excessive cross-linking of dityrosine. From Table S1, it can be found that the exposure time under white light has little effect on the compressive properties of BSA DC gel. Nevertheless, the exposure time distinctly influenced the mechanical properties of BSA CC gel, and it was difficult for it to gain enough strength to be removed from the mold if the exposure time was <4 h. Therefore, all of the BSA DC gels were prepared under exposure for 4 h. Moreover, the denaturation temperature and time also affected the compressive properties. BSA DC gel achieved the best compressive strength at a heating temperature of 80°C and a heating time of 10 min. Unless otherwise stated, we mainly focus on the BSA DC gel prepared at optimal conditions, i.e., C_BSA_ of 500 mg/mL, C_Ru(II)_ of 600 μM, exposure time of 4 h, heating temperature of 80°C, and heating time of 10 min, which achieved σ_c_ of 37.81 ± 2.61 MPa and E_c_ of 1515 ± 43 kPa.

### Rheology

**Figure 4:**
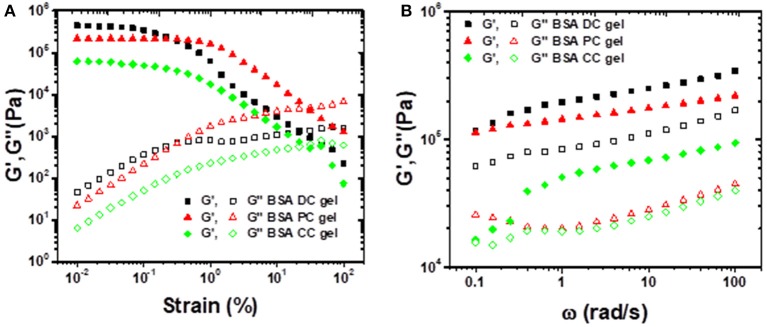
**(A)** Strain sweep experiments performed from 0.01 to 100% strain under a frequency of 10 rad/s; **(B)** frequency sweep experiments performed from 0.1 to 100 rad/s under a shear strain of 0.1%.

Rheological tests were also conducted to evaluate the viscoelastic properties of BSA gels. As shown in Figure 4A, under low shear strain (γ ≤ 1%), all the three BSA gels exhibited a plateau without a change in storage modulus (*G*′), however, *G*′ deceased along with shear strain as γ > 1%. In contrast, the loss modulus (*G*″) was perpetually increased with an increase in shear strain. Clearly, at γ ≤ 1%, *G*′ of BSA DC gel was 4,457 Pa, which was greater than those of BSA PC gel (2167 Pa) and BSA CC gel (627 Pa). We estimated the effective network chain density (*N*_1_) of the gels by *G*_*e*_ = *N*_1_*RT*, where *G*_*e*_, *R*, and *T* are the equilibrium shear modulus, gas constant, and absolute temperature, respectively. Taking the room temperature (~25°C) as the reference temperature, the estimated *N*_1_ values were 172, 85, and 24 mol/m^3^ for BSA DC gel, BSA PC gel, and BSA CC gel, respectively. Distinctly, the results indicate that BSA DC gel demonstrates much higher cross-linking density compared to the other two gels, which was consistent with the morphological results in Figure 2. Moreover, compared to BSA CC gel, *N*_1_ for BSA DC gel increased 7.2 times, inferring that the physical cross-linkings induced by heat denaturation of BSA were dominant in BSA DC gel. In Figure 4B, all the three gels can be seen to show ω-dependent behaviors, and *G*′ and *G*″ increased as ω increased, indicating that they displayed viscoelasticity. In addition, *G*′ was always larger than *G*″ for the three BSA gels, implying that they exhibit elastic properties under small deformation (γ = 0.1%).

### Non-swelling Properties

**Figure 5:**
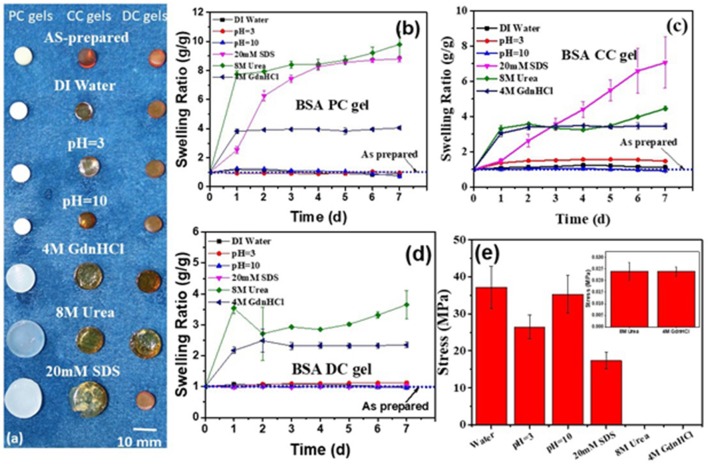
**(a)** Photographs of three BSA gels after swelling in different solutions (inset reveals magnified plot in 8 M urea and 4 M GdnHCl solution; the scale is 10 mm); the swelling ratio in different solutions of BSA PC gel **(b)**, BSA CC gel **(c)**, and BSA DC gel **(d,e)** compressive strength of BSA DC gels after swelling in various solutions.

The stability of hydrogel in aqueous solution is a key parameter for their potential applications. Therefore, the swelling of the three BSA gels in various solvents was investigated (Figure 5). Photographs of the three BSA gels at the as-prepared and swollen state are presented in Figure 5a. Except for BSA CC, which was gel slightly swollen in pH = 3, all of the three BSA gels demonstrated negligible size change in DI water, pH = 3 and pH = 10, indicating non-swelling properties in these solutions. However, in 8 M urea and 4 M guanidine hydrochloride (GdnHCl) solution, all three BSA gels had an obvious size increase, indicating that the gels swelled significantly in the two solutions. It is well-known that urea and GdnHCl are classical denaturants for proteins. The swelling of the BSA gels indicates that there is unfolding of BSA in these gels. The gels differed, however, in that BSA DC gel was non-swollen in sodium dodecyl sulfate (SDS) solution, while BSA PC gel and BSA CC gel swelled distinctly in SDS solution. To quantitatively evaluate the swelling properties of BSA gels, the weights of swollen and as-prepared gels were measured. The swelling kinetics of the three BSA gels are illustrated in Figures 5b–d. Consistently, the three BSA gels were almost unswelling in DI water, pH = 3, and pH = 10. All three BSA gels swelled substantially in 8 M urea and 4 M GdnHCl solution. Specifically, BSA DC gel had the largest swelling ratio in 8 M urea and 4 M GdnHCl solution after swelling for 7 days, i.e., 3.65 g/g and 2.35 g/g, respectively; however, it was an exception in being unswollen in SDS solution. The non-swelling mechanism of BSA DC gels is still unclear. Probably, it is a feature of the BSA biomacromolecule. As shown in Figures 5a–d, regardless of physical or chemical cross-linkings, BSA PC gels and BSA CC gels are not swollen in DI water, pH = 3.0, and pH = 10.0. However, in denaturant solution, both of them are swollen significantly. The results indicate that BSA is further unfolded in the denaturant solution, while it maintained its conformation in DI water and at various pH values. Therefore, BSA DC gels are also non-swollen in water with various pHs. However, the synergistic effect of physical and chemical cross-linkings may also play an important role in suppressing the unfolding of BSA in SDS solution. Therefore, BSA PC gel and BSA CC gel swell markedly in SDS solution, while BSA DC gel is unswelling. The compressive properties of BSA DC gel after swelling are illustrated in Figure 5e. After swelling, the σ_c_ values of BSA DC gel in DI water, pH = 3.0, pH = 10.0, and 20 mM SDS solution were 35.85 ± 5.66, 23.60 ± 3.21, 30.27 ± 5.17, and 17.34 ± 2.23 MPa (Figure 5e, Figure S2 and Table S2), respectively. However, the σ_c_ values were only 0.028 ± 0.004 and 0.025 ± 0.002 MPa after swelling in 8 M urea and 4 M GdnHCl, respectively. The results indicate that our BSA DC gels are unswollen in water and maintain good compressive properties after swelling in water.

### Hysteresis, Self-Recovery, and Fatigue Resistance

**Figure 6:**
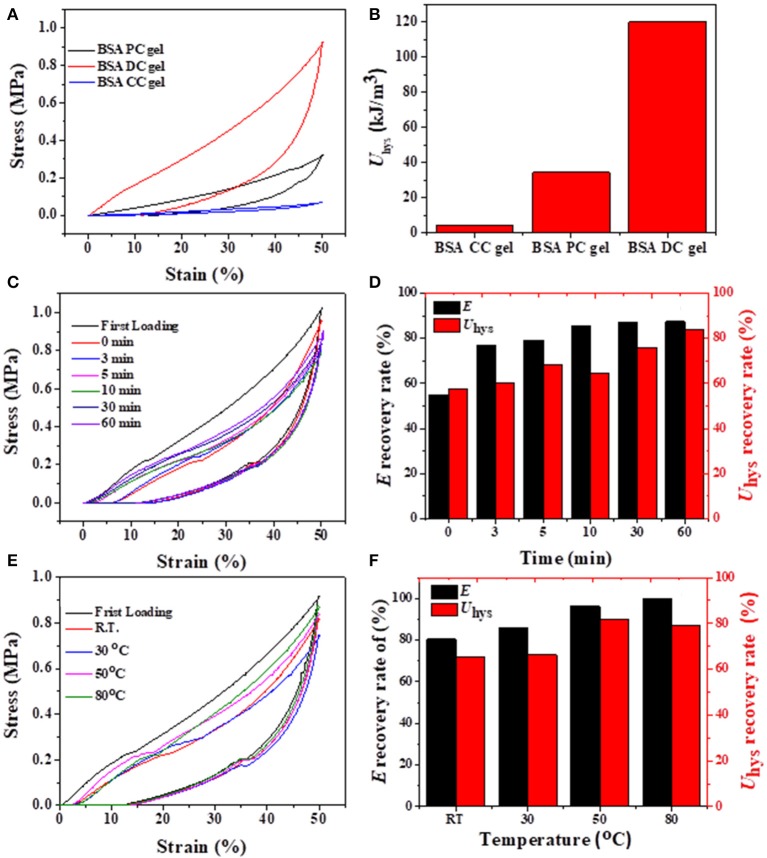
**(A)** Loading-unloading curves of three BSA gels and **(B)** corresponding dissipated energies; **(C,D)** recovery of BSA DC gel at RT for various times; **(E,F)** recovery of BSA DC gel at various temperatures with 5 min recovery time between two consecutive loading cycles.

Cyclic loading-unloading compressive tests were performed to evaluate the energy dissipation capacity of BSA DC gels. As shown in Figure 6A, BSA CC gel showed negligible hysteresis, while both BSA PC gel and BSA DC gel exhibited large hysteresis loops at a compressive strain of 50%. Moreover, the hysteresis loop of BSA DC gel was much larger than that of BSA PC gel. Specifically, the dissipated energies of BSA DC gel, BSA PC gel, and BSA CC gel were 120.04, 34.46, and 4.23 kJ/cm^3^ (Figure 6B), respectively. The results indicate that BSA DC gel exhibits much larger energy dissipation ability, which is the reason why BSA DC gel demonstrates higher compressive strength than the other two BSA gels. The hysteresis loops also increased with increasing compressive strain, and *U*_hys_ increased from 2.36 to 624.75 kJ/m^3^ as compressive strain increased from 10 to 80% (Figures S3A,B). Besides, successive loading-unloading without an interval between two consecutive loading cycles for BSA DC gel was also performed (Figures S3C,D). The hysteresis loops also increased with the increase in compressive strains, and distinct overlap was detected between two consecutive loading cycles, indicating that BSA DC gel partially recovers during the unloading. Therefore, the recovery behavior of BSA DC gel at room temperature (RT) for various recovery times was investigated (Figures 6C,D), and it was found that the hysteresis loops became larger when the recovery time was longer. Two recovery rates, i.e., stiffness recovery (based on *E*) and toughness recovery (based on *U*_hys_), were defined as in our previous reports (Chen et al., 2017). Without recovery time, the stiffness and toughness of BSA DC gel were recovered to 55 and 58%, respectively. Impressively, with recovery for only 3 min, the recovery rates for stiffness and toughness reached up to 77 and 60%, respectively. At a recovery time of 60 min, stiffness recovery and toughness recovery were 87 and 84%, respectively. The effect of recovery temperature on the recovery of BSA DC gels was also investigated (Figures 6E,F). The hysteresis loops also increased with the increase in recovery temperature, and stiffness recovery and toughness recovery reached up to 100 and 87% at 80°C, respectively. The results indicate that our BSA DC gels demonstrated excellent and rapid self-recovery properties.

**Figure 7:**
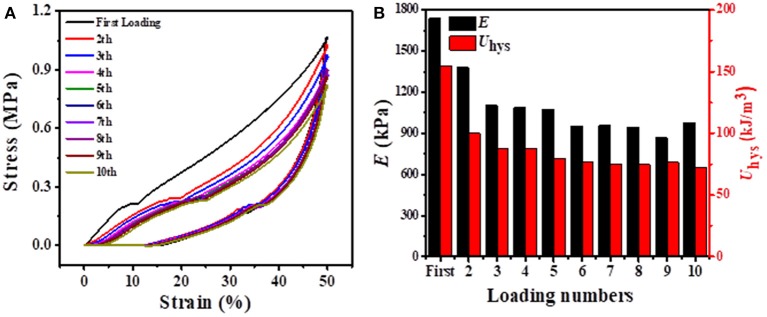
**(A)** 10 successive loading-unloading cycles for the same BSA DC gel with 3 min recovery time between two consecutive loading cycles at a compressive strain of 50%; **(B)** corresponding elastic modulus and dissipated energy with loading cycling.

Considering that our BSA DC gels displayed excellent self-recovery properties at RT, they might also show fatigue resistance. As shown in Figure 7A, 10 successive loading-unloading cycles for the same BSA DC gel demonstrated that the hysteresis loops became the same after several loading cycles. Specifically, *E* and *U*_hys_ were approximately 908 kPa and 74 kJ/m^3^ after several loading cycles (Figure 7B), respectively. Moreover, the BSA DC gel maintained integrity without breakage after the 10th loading cycle (data not shown). The results indicate that our BSA DC gels also have excellent fatigue resistance.

## Conclusion

In summary, BSA DC gels, featuring hybrid chemical and physical cross-linkings, were prepared by a facile photoreaction-heating process. Owing to the hybrid cross-linkings, BSA DC gels demonstrated high strength, large hysteresis, rapid self-recovery, and excellent fatigue resistance at room temperature. The compressive strength of BSA DC gels was 37.81 ± 2.61 MPa, which was comparable to articular cartilage. More interestingly, BSA DC gel displayed high transparency and non-swelling properties while maintaining its high performances in DI water, pH = 3.0, and pH = 10.0. With a combination of simple preparation, high strength, and non-swelling properties, it is believed that BSA DC gels can potentially be applied in biomedical fields.

## Experimental Section

### Materials

Ammonium persulfate (APS, ≥98%) was purchased from Aladdin (Shanghai), and Bovine serum albumin (BSA, 98%) and tris(2,2′-bipyridine) ruthenium(II) dichloride [Ru(II)] were purchased from Sigma-Aldrich Inc. All materials were used without further purification.

### Preparation of BSA DC Gels

BSA DC gels were prepared by a photoreaction-heating process as reported. Briefly, BSA (500 mg/mL), APS (300 mM), and Ru(II) (600 μM) were first dissolved into DI water. The precursor solution obtained was then injected into a cylindrical mold (D = 8.5 mm) and was exposed to white light (at a power of 8 W) for 4 h. The gel was then heated in a water bath (60–90°C) for 0–30 min. After cooling to room temperature, BSA DC gels were stored at 4^o^C for testing. BSA CC gels were synthesized via photoreaction without heating, while BSA PC gels were fabricated via heating pure BSA solution [without APS or Ru(II)] at 80°C for 10 min.

### Characterizations

#### Mechanical Tests

Compressive and tensile tests were carried out with a WSM-10 kN machine (Changchun Zhineng Instrument Company). For compressive tests, the cylindrical gel samples (diameter = 8.5 mm, height = 10 mm) were conducted at 5 mm/min. The compressive stress (σ_c_) was defined as F/A_0_, where F is the force applied to the sample and A_0_ is the initial cross-sectional area of the sample. The compressive strain was estimated as h/h_0_, where h is the height of deformed gel and h_0_ is the original height. The uniaxial tests of BSA DC gels (thickness = 1 mm and length = 60 mm) were pulled up at a constant rate of 100 mm/min. The tensile stress and strain were measured as F′/A_0_^′^ and l/l_0_, where F′ is the force applied to the sample, ${\text{A}}_{0}^{\prime }$ is the initial cross-sectional area of the sample, l is the length of the stretched specimen, and l_0_ is the original length. The compressive and tensile moduli were calculated from the slope of the linear region of the stress-strain curves. At least three independent experiments were conducted. The errors were calculated via Excel. The arithmetic mean value of the data was calculated first, and then the standard deviation (S.D.) was calculated. Statistical analysis was done by one-way analysis of variance (ANOVA) and LSD's multiple comparison post-test with a 99.9% confidence interval among the applied treatments (*p* < 0.01). Cyclic loading tests of gels were also performed by the abovementioned machine at 5 mm/min with maximum strain from 10 to 80%. Successive loading-unloading tests were done on the same gel specimen. For self-recovery tests, the samples were recovered at different temperatures (RT to 80°C) for various times (0–60 min). The dissipated energies were calculated via the area between loading-unloading curves. The recovery rates were defined as the rate of dissipated energy (*U*_hys,t_) or elastic modulus (*E*t) to the first loading cycle (*U*_hys,0_ or *E*_0_). Stiffness recovery was defined as the ratio of the elastic modulus at various times or temperatures to that of the first loading cycle, while toughness recovery was evaluated as the ratio of dissipated energies at various times or temperatures to those of the first loading cycle at a maximum compressive strain of 50%.

#### Swelling Tests

As-prepared hydrogels (diameter = 8.5 mm, height = 10 mm) were firstly weighed and then immersed in different solutions at 25°C. After a predetermined time, the swollen hydrogels were taken out and weighed again. The swelling ratio (SR) was obtained from the following equation: SR = m_t_/m_0_, where m_0_ and m_t_ are the weight of as-prepared gel and swollen gel, respectively.

#### Other Tests

Scanning electron microscope (SEM) tests were performed using a MERLIN Compact instrument (Zeiss, Germany) at a voltage of 15 KV. All the gel samples were frozen and fractured in liquid nitrogen, and they were then dried in a lyophilizer. Before the tests, the samples were coated with a thin layer of gold. Fourier transform infrared spectra (FT-IR) of the BSA hydrogel powders were obtained with a Bruker Tensor 27. The BSA gel sheet was cut into a disk for rheological tests (thickness = 1 mm, diameter = 25 mm). Rheological tests were carried out on a rheometer (Anton Paar, MCR302) via the plate-plate mode. Amplitude sweep tests were carried out at ω of 10 rad/s and 25°C. Frequency sweep tests were performed at a strain of 0.1%, ω of 0.1–100 rad/s, and at 25°C. The ultraviolet spectrum of BSA DC gel was recorded on a double-beam UV-visible spectrophotometer (General Analytical Instruments Co. Ltd, TU-1901, Beijing).

## Data Availability Statement

The raw data supporting the conclusions of this article will be made available by the authors, without undue reservation, to any qualified researcher.

## Author Contributions

The manuscript was prepared by QC and GS, and the gels were synthesized by SL, LZ, and QW. The tests were cooperated and finished by SL, ZL, CT, and HS. The data were analyzed by JY, GQ, and GS.

### Conflict of Interest

The authors declare that the research was conducted in the absence of any commercial or financial relationships that could be construed as a potential conflict of interest.
