# Supplementary material for: High-Strength Albumin Hydrogels With Hybrid Cross-Linking
Source: Front Chem. 2020 Feb 25;8:106. doi: 10.3389/fchem.2020.00106 (PMC7052378; doi:10.3389/fchem.2020.00106)
Supplement: Supplementary file 1 [file Data_Sheet_1.docx]

# Supporting Information

**High Strength Albumin Hydrogels with Hybrid Cross-Linking**

Shaoping Lu^1, #^, Lin Zhu^1, #^, Qiling Wang^1^, Zhao Liu^1^, Cheng Tang^1^, Huan Sun^1^, Jia Yang^1^, Gang Qin^1^, Gengzhi Sun^2^, Qiang Chen^1,^ *

1. School of Materials Science and Engineering, Henan Polytechnic University, Jiaozuo, 454003, China.
2. Key Laboratory of Flexible Electronics (KLOFE) & Institute of Advanced Materials (IAM), Jiangsu National Synergetic Innovation Center for Advanced Materials (SICAM), Nanjing Tech University (NanjingTech), Nanjing, China, 211816

**Figure S1**. Peak at 460 nm of UV-visible spectroscopy

**Figure S2.** Compression stress-strain curves of BSA DC gels after swelling in various solutions

**Figure S3.** a) Cyclic Loading-unloading curves of BSA DC gels strain from 10% to 80% (inset reveals magnified plot in low-strain region) and b) the dissipated energy (inset reveals magnified plot in low-strain region); c) Successive loading-unloading curves of BSA DC gel under different strains (inset reveals magnified plot in low-strain region) and d) the corresponding dissipated energy (inset reveals magnified plot in low-strain region)

**Table S1.** Effect of various parameters on the compressive properties of BSA DC gels.

| Entry | C_BSA_ (mg/mL) | C_Ru(II)_  (μM) | exposure time(h) | heating temperature(℃) | heating time(min) | *ε*_c, f_  （%） | *σ*_c_  (MPa) | *E*  (kPa) |
| --- | --- | --- | --- | --- | --- | --- | --- | --- |
| 1 | 100 | 600 | 2 | 80 | 10 | -- | --^a^ | --^a^ |
| 2 | 200 | 600 | 2 | 80 | 10 | 63.75±0.83 | 0.54±0.03 | 292±17 |
| 3 | 300 | 600 | 2 | 80 | 10 | 79.64±0.38 | 3.22±0.61 | 512±8 |
| 4 | 400 | 600 | 2 | 80 | 10 | 88.49±0.58 | 26.00±2.10 | 832±17 |
| 5 | 500 | 600 | 2 | 80 | 10 | 88.84±0.59 | 34.73±2.92 | 1320±25 |
| 6 | 500 | 0 | 2 | 80 | 10 | 83..93±0.29 | 3.10±0.06 | 253.5±8 |
| 7 | 500 | 200 | 2 | 80 | 10 | 88.98±0.23 | 23.45±1.37 | 507±21 |
| 8 | 500 | 400 | 2 | 80 | 10 | 89.18±0.27 | 27.90±1.45 | 1065±52 |
| 9 | 500 | 600 | 2 | 80 | 10 | 88.84±0.59 | 34.73±2.92 | 1320±25 |
| 10 | 500 | 800 | 2 | 80 | 10 | 87.09±1.48 | 35.85±2.10 | 1355±34 |
| 11 | 500 | 1000 | 2 | 80 | 10 | 85.05±5.96 | 36.08±3.08 | 1471±78 |
| 12 | 500 | 600 | 1 | 80 | 10 | 85.76±0.80 | 30.70±0.65 | 1710±131 |
| 13 | 500 | 600 | 2 | 80 | 10 | 85.21±1.45 | 32.73±4.95 | 1584±77 |
| 14 | 500 | 600 | 3 | 80 | 10 | 84.96±0.89 | 34.66±1.84 | 1567±14 |
| 15 | 500 | 600 | 4 | 80 | 10 | 86.16±0.96 | 37.81±2.61 | 1515±43 |
| 16 | 500 | 600 | 4 | 60 | 10 | 88.25±0.18 | 24.68±2.53 | 470±44 |
| 17 | 500 | 600 | 4 | 70 | 10 | 89.02±0.41 | 35.73±2.71 | 993±58 |
| 18 | 500 | 600 | 4 | 80 | 10 | 88.02±0.33 | 36.83±2.66 | 1583±43 |
| 19 | 500 | 600 | 4 | 90 | 10 | 83.59±0.18 | 21.84±1.08 | 1707±87 |
| 20 | 500 | 600 | 4 | 80 | 0 | 77.88±1.68 | 2.49±0.29 | 284±48 |
| 21 | 500 | 600 | 4 | 80 | 1 | 81.74±2.23 | 10.69±2.60 | 358±42 |
| 22 | 500 | 600 | 4 | 80 | 5 | 81.58±1.37 | 16.44±3.42 | 1568±7 |
| 23 | 500 | 600 | 4 | 80 | 10 | 86.16±0.92 | 37.08±2.61 | 1515±43 |
| 24 | 500 | 600 | 4 | 80 | 20 | 81.78±0.98 | 22.68±1.94 | 2521±79 |
| 25 | 500 | 600 | 4 | 80 | 30 | 83.07±0.51 | 22.61±0.48 | 2625±116 |

Table S2. Mechanical properties BSA DC gel in different solutions

| samples | Water | pH=3 | pH=10 | 20mM SDS | 8M Urea | 4M GdnHCl |
| --- | --- | --- | --- | --- | --- | --- |
| *ε*_c, f_ (%) | 88.45±1.25 | 87.85±1.78 | 87.58±1.65 | 84.58±1.38 | 26.77±3.12 | 27.88±4.18 |
| *σ*_c_ (MPa) | 35.85±5.66 | 23.60±3.21 | 30.27±5.17 | 17.05±2.23 | 0.028±0.004 | 0.025±0.002 |
| *E* (kPa) | 1100±107 | 1027±81 | 1167±45 | 1116±56 | 59±5 | 53±8 |
